# Anti-predator defences are linked with high levels of genetic differentiation in frogs

**DOI:** 10.1098/rspb.2023.2292

**Published:** 2024-01-24

**Authors:** Iliana Medina, Caroline Dong, Roberto Marquez, Daniela M. Perez, Ian J. Wang, Devi Stuart-Fox

**Affiliations:** ^1^ School of BioSciences, University of Melbourne, Melbourne 3010, Australia; ^2^ Department of Ecology and Evolutionary Biology, Tulane University, New Orleans, LA 70115, USA; ^3^ Department of Ecology and Evolutionary Biology and Michigan Society of Fellows, University of Michigan, Ann Arbor, MI 48109, USA; ^4^ Max Plank Institute of Animal Behaviour, 78464 Konstanz, Germany; ^5^ Department of Environmental Science, Policy, and Management, Rausser College of Natural Resources, University of California, Berkeley, CA 94720, USA

**Keywords:** aposematism, gene flow, speciation, divergence, frogs

## Abstract

Predator–prey interactions have been suggested as drivers of diversity in different lineages, and the presence of anti-predator defences in some clades is linked to higher rates of diversification. Warning signals are some of the most widespread defences in the animal world, and there is evidence of higher diversification rates in aposematic lineages. The mechanisms behind such species richness, however, are still unclear. Here, we test whether lineages that use aposematism as anti-predator defence exhibit higher levels of genetic differentiation between populations, leading to increased opportunities for divergence. We collated from the literature more than 3000 pairwise genetic differentiation values across more than 700 populations from over 60 amphibian species. We find evidence that over short geographical distances, populations of species of aposematic lineages exhibit greater genetic divergence relative to species that are not aposematic. Our results support a scenario where the use of warning signals could restrict gene flow, and suggest that anti-predator defences could impact divergence between populations and potentially have effects at a macro-evolutionary scale.

## 1. Introduction

Animals and plants have evolved an incredible diversity of mechanisms to avoid predation, and various studies have linked the presence of anti-predator defences in some lineages to their evolutionary success. Ehrlich and Raven [1] proposed, for instance, that the evolution of novel defences against herbivory allowed some plants to ‘radiate’ and diversify into new niche space. The high diversity of some plant lineages could be explained by lineages escaping the costs of predation (known as the *escape and radiate* hypothesis). Although this specific example has received mixed support [2], other studies point to similar scenarios. For example, venomous families of insects and fish have diversification rates twice as high as non-venomous families [3]. Similarly, the blenny fish genus *Meiacanthus* has buccal venom glands as defence and higher diversification rates compared with closely related lineages that lack venom [4]. Hence, accumulating evidence suggests that strong anti-predator defences may lead to faster diversification, but the mechanisms through which anti-predator defences contribute to speciation remain poorly understood.

Animals with warning signals employ bright colour combinations to advertise toxicity or unpalatability to potential predators [5], and this anti-predator strategy, known as aposematism, has evolved in many lineages. Aposematism decreases predation because predators quickly learn to avoid prey displaying warning signals [5,6]. Several studies suggest that aposematic lineages could have higher species richness compared with non-aposematic lineages [7,8]. More specifically, in amphibians the acquisition of conspicuous coloration in toxic species is associated with high diversification rates [9]. Within frogs, it has also been shown that aposematic poison frogs of the family Dendrobatidae have higher speciation rates than non-aposematic lineages in the same family [10]. There is considerable evidence that aposematism could be linked with higher speciation rates; however, there is little known about the micro-evolutionary mechanisms driving the link between aposematism and increased rates of speciation.

Previous studies have suggested that ecological opportunity due to decreased predation could have resulted in increased diversification in protected lineages. Namely, the use of aposematism as an anti-predator strategy could result in a reduction in hiding behaviours, which could lead to a greater use of opportunities across space [9,11]. Another possibility is that aposematic lineages have higher diversification (and higher speciation rates) because there is lower gene flow between populations, which could accelerate divergence. Across a wide variety of taxa, lineages with higher speciation rates tend to exhibit low levels of gene flow between populations. For example, birds with higher speciation rates have smaller wings, low dispersal abilities and higher population differentiation [12,13]. Likewise, the loss of flight in beetles is linked with both higher genetic differentiation among populations and higher speciation rates [14]. Hence, one possible explanation for the high speciation rates reported in aposematic lineages is that they have more restricted gene flow between populations (compared with other lineages), which could facilitate speciation [15,16]. However, there are not yet any studies with formal tests of this hypothesis (as far as we are aware).

How could aposematism restrict dispersal and gene flow? This anti-predator strategy is positively density dependent, and it is widely accepted that the efficiency of a warning signal increases steadily with its local frequency in the environment [17–19]. High abundance of aposematic prey decreases the risk of being attacked by a predator, and the costs of predator training are shared [16,20]. This is also a form of Allee effect, where there is decreased fitness at low population size or density. Allee effects are known to reduce dispersal distance of organisms [21,22] and are also linked with high genetic diversity [23]. Aposematism could potentially restrict gene flow between populations if there is selection for reduced dispersal due to Allee effects (because colonizing new environments would be harder due to naive predators) or if locally trained predators select against migrants with divergent phenotypes in established populations. Restricted dispersal in aposematic lineages could then facilitate divergence between populations and ultimately speciation.

In this study, we use a meta-analytical approach to test whether aposematism is associated with increased genetic divergence between populations and whether this pathway could explain the high diversification rates found in aposematic lineages. We collated published information on genetic differentiation between pairs of populations of amphibians and tested whether species that are aposematic have higher levels of genetic differentiation (i.e. lower levels of gene flow) compared with species that are not aposematic. Our study offers a link between macro-evolutionary patterns previously reported and micro-evolutionary mechanisms associated with predator–prey interactions.

## 2. Methods

### (a) Systematic literature search

We searched ISI Web of Science and Scopus on 13 April 2020 for peer-reviewed, English language studies that measured some proxy of genetic differentiation (e.g. *F*_ST_ or *G*_ST_) and geographical distance (i.e. GPS coordinates) for amphibians (electronic supplementary material, figure S1). *F*_ST_ is the main measure of genetic structure used in the literature and provides the greatest sample size for a meta-analysis. Although there is debate around the accuracy of *F*_ST_ as a measure of genetic structure [24], it is the most widely used method and still considered to be a valid and accurate measure of genetic differentiation under a broad range of conditions [25–27]. A detailed list of search terms is given in the electronic supplementary material, but broadly, we looked for studies with the following words: (‘genet*’ OR ‘genetic diff*’ OR ‘population structure’ OR ‘gene flow’ OR ‘dispersal’ OR ‘phylog*’ OR ‘landscape genetic*’) AND (‘Fst’ OR ‘Gst’ OR ‘D’ OR ‘F’ OR ‘F st’ OR ‘G st’) AND (‘amphibia*’ OR ‘frog*’ OR ‘salamand*’ OR ‘toad*’).

After removing duplicate papers recovered from both Web of Science and Scopus, we read the titles and abstracts of the remaining 1327 papers. We removed papers that were not relevant because they were not about population genetics or about amphibians. This left 532 papers, for which we read the full text and selected studies that reported values of genetic differentiation *within* species, given our focus on differentiation is at this level. There were 225 studies left, for which we aimed to extract pairwise values of genetic differentiation (in *F*_ST_, *G*_ST_), geographical coordinates associated with each of the populations, species studied, average sample size per population, type of genetic marker used in analysis (microsatellites, mitochondrial DNA (mtDNA), single nucleotide polymorphisms (SNPs) or other) and the type of estimator used (*F*_ST_, *G*_ST_). We were able to extract or obtain the relevant information for 89 studies, since several studies did not calculate pairwise *F*_ST_ values between populations (e.g. just provided a global *F*_ST_ for the species). If microsatellites were employed, we also recorded information on the number of loci used. In those cases where the tables of genetic differentiation values or geographical coordinates were not publicly accessible (but were calculated), we contacted the authors of the publication. We obtained the coordinates for all studies, except for two, which did not provide coordinates (or a map where these could be extracted from) but provided a matrix of geographical distances. All coordinates extracted were converted to decimal system and then imported to calculate topographic distances (described below).

To maximize our sampling of aposematic species (which are rarer), we calculated the *F*_ST_ matrices for three studies that published appropriate genetic data but had not calculated or reported pairwise *F*_ST_ matrices. For Rabemananjara *et al.* [28], we downloaded mtDNA data from GenBank (accessions DQ889341-DQ889429), aligned them using Muscle Edgar [29], and used the *pairwise_Gst_Nei* function from the mmod R package to calculate Nei's *G*_ST_ estimator [30]. For Lawrence *et al.* [31] and Márquez *et al.* [32], we downloaded vcf files from each study's data repository and used vcftools [33] to calculate Weir & Cockerham's [34] *F*_ST_ estimator, averaged across sites as a ratio of averages (see Bhatia *et al.* [35]). To reduce biases due to linkage disequilibrium, only sites at least 1 kb apart were used.

To explore whether there were signs of publication biases in our dataset we followed Gandra *et al.* [36] and visually inspected the distribution of *F*_ST_ values, expecting it to be unimodal and decreasing towards higher values of differentiation (given that all values were calculated within species). We also tested whether there was any association between the sample size of a study (the number of individuals sampled per population) and the *F*_ST_ values calculated.

#### (i) Extraction of topographic geographical distances

To estimate biologically relevant geographical distances between the pairs of populations with available genetic differentiation information, we used the recently developed R package topoDistance [37]. The function *topoDist* employs elevation rasters, which we acquired using the package elevatr [38], and calculates distances while accounting for the additional distance imposed by topographic relief. In this way, the distances calculated capture the entire distance along the path an organism must move between two geographical locations, which is important for non-flying organisms. We used the function *get_elev_raster* to extract the raster for each set of locations per study and a zoom of 10, corresponding to approximately 75–150 m resolution (except when memory was exhausted and zoom was reduced to 7). Then, the function *topoDist* generated a square matrix with all of the topographic distances between pairs of locations.

#### (ii) Additional variables

Information on whether a species was considered aposematic or not was extracted from Arbuckle & Speed [9]. In this study, species were classified as having chemical defences (yes/no) and as being conspicuous (yes/no). We considered only those species that were both conspicuous and chemically defended as aposematic species. Seven species did not have information on chemical defences, so for these we inferred toxicity based on the most closely related species with available information (details shown in the electronic supplementary material, dataset). Given that larger species might have different dispersal abilities from smaller species [39,40], we also collected information on body size (snout–vent length in mm) from different sources (mainly Oliveira *et al.* [41], specified in dataset).

#### (iii) Statistical analyses

We built a matrix with all pairwise comparisons from all included studies. Genetic differentiation values were *F*_ST_ in all cases (other estimates were also reported in a few studies, but we only used *F*_ST_ because it was far more common). Negative values were transformed to 0.001 and maximum values to 0.9999. We used the formula *F*_ST_/(1−*F*_ST_) to linearize the *F*_ST_ values, following Slatkin [42], and then used a logarithmic transformation. This process is equivalent to a logit transformation, which is commonly used and facilitates model convergence for this type of data [25–27,36,43]. Topographic distances were also log-transformed.

To test whether aposematic organisms accrue greater genetic differentiation than non-aposematic organisms, we built a generalized linear mixed model (GLMM) using the package MCMCglmm [44]. The response variable was the logit transformation of *F*_ST_ for each pairwise comparison, and we used as predictors of genetic differentiation (i) the topographic distance (log) between the two populations, (ii) whether the species is considered aposematic or not, (iii) the body size of the species (in mm), and (iv) the type of genetic marker employed for the *F*_ST_ calculation, and the interaction between distance and anti-predator strategy (aposematic or not). An effect of anti-predator status or its interaction with distance would suggest that there are differences in the levels of genetic differentiation achieved between both categories. As random terms in the model we used the species identity and the study reference, given that some species were included in different studies and some studies included multiple species. We considered differences in sample size between studies by adding a weighting argument (mev) to the models, where studies with higher sample sizes per population were weighted higher in the model. To account for phylogenetic relationships as well as phylogenetic uncertainty, we downloaded a distribution of 1000 trees randomly sampled from the posterior distribution of the analyses in Jetz & Pyron [45]. For our dataset, all species except for one had phylogenetic data. Phylogenetic relationships between species were considered by adding a random term in the model, using a distance matrix calculated from a phylogenetic tree (from different phylogenetic hypotheses).

The main model described above was run following Ross *et al.* [46]. Briefly, we ran the model using 1000 trees and for each tree used 1500 iterations, saving only the last iteration before going into the next tree and repeating the process. We used the first 100 iterations (100 trees) as burnin and assessed model convergence, ensuring that the effective sample size was always above 800. In addition to the main model, we also examined a slight variation of the model because of the possibility that relationships between distance and *F*_ST_ are not completely linear. To account for this possibility, we used a generalized additive mixed model (GAMM) in the R package *brms* [47], which fits a smooth function to predict values of the response variable (logit *F*_ST_ in our case). We used as predictors a smooth function with an interaction term for log distance and aposematic status (in the form s(log distance, aposematic status, bs = ‘fs’)). We only used the GAMM model for the microsatellite dataset, which included the majority of studies (69% of data), because the model did not converge when the full dataset was used. We added as random effects the species identity, study ID and the phylogenetic structure matrix. We use this model mainly as a visual aid, given that the interpretation of the statistical test is centred around testing whether the slope of the whole smooth function is different from zero or not (which it is for both categories). Given that in the GAMM visualization we noticed that the linear relationship between *F*_ST_ and distance was maintained only up to a certain genetic distance, we also performed a GLMM as described initially but using a reduced dataset (details in Results).

Since the distribution of the sampled aposematic species could be biased towards the tropics (i.e. Dendrobatidae is a tropical family), and given that species in the tropics might have higher divergence rates [48,49], we also fit models to consider the possible effect of latitude on genetic divergence. These models are described in detail in the electronic supplementary material, table S5. In addition to these tests, we also fitted an additional model where we divided non-aposematic species into species that are chemically defended and species that are not (based on data from Arbuckle & Speed [9]). This generated three categories: aposematic species (conspicuous and chemically defended *n* = 21), toxic species (non-conspicuous but chemically defended *n* = 29) and non-toxic and non-conspicuous species (*n* = 14). Unfortunately, our literature search did not identify any suitable data for non-toxic, conspicuous species, so there were none of these species included in our dataset (and only 2% in Arbuckle and Speed's original dataset). We used the same model structure as in previous models to test whether there were differences in genetic differentiation between these three categories of anti-predator defence. Given the smaller sample size in each category, we do not focus our discussion around this model.

Finally, to test whether our main results were robust to biases due to the effect of specific populations, or to studies with high number of populations, we also used a randomization procedure (described in the electronic supplementary material, figure S3). We also confirmed that the aposematic species in our dataset presented higher speciation rates compared with the non-aposematic species sampled, using recently published tip-speciation rates for anurans [50] (electronic supplementary material, figure S4).

## 3. Results

**Figure 1.**
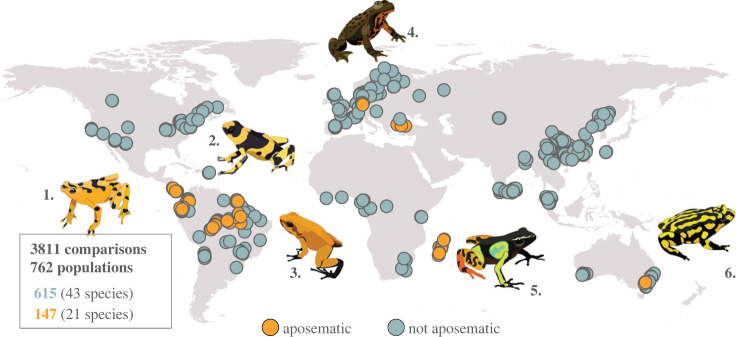
Distribution of distinct populations included in the analyses (only anurans). For two studies we had information on geographic distances but not geographic coordinates, so these populations are not included in the figure. Drawings show representative aposematic species in our dataset: 1. *Atelopus zeteki*, 2. *Oophaga lehmanni*, 3. *Phyllobates terribilis*, 4. *Bombina variegata*, 5. *Mantella baroni*, 6. *Pseudophryne corroboree*. Drawings by D.M.P.

We were able to extract complete information on geographical and genetic distances for 5365 pairs of populations, representing 89 different studies and 74 different species. From these studies, 14 corresponded to salamanders but there was only one salamander species considered non-aposematic, so we decided to focus our analyses on anurans (64 species in total, 75 independent studies, 762 populations, 3811 pairwise comparisons, figure 1). Within frogs, we obtained information for 21 aposematic species and 43 non-aposematic species. Aposematic species belonged to the families Dendrobatidae (11 spp.), Mantellidae (5 spp.), Bufonidae (2 spp.), Myobatrachidae (1 sp.) and Bombinatoridae (2 spp.). Non-aposematic species belonged to the families Leptodactylidae (1 spp.), Hylidae (6 spp.), Arthroleptidae (1 sp.), Ascaphidae (1 sp.), Bufonidae (9 spp.), Ranidae (13 spp.), Eleutherodactylidae (2 spp.), Limnodynastidae (1 sp.), Heleophrynidae (1 sp.), Dicroglossidae (1 sp.), Ranixalidae (1 sp.), Pelobatidae (1 sp.), Rhacophoridae (2 spp.), Megophryidae (1 sp.) and Craugastoridae (2 spp.).

We found that the range of topographic distances for aposematic species was slightly lower than for non-aposematic species, and there were fewer populations separated by long distances in the set of aposematic species. The maximum distance between populations was 851 km for aposematic species and 3982 km for non-aposematic species (electronic supplementary material, figure S2*a*), and although the distributions of distances are slightly positively skewed, they both appear to be unimodal. The distribution of *F*_ST_ values was unimodal and skewed towards lower values (electronic supplementary material, figure S2*b*), suggesting there is no obvious bias towards publishing studies with values of high genetic differentiation. We found an association between a study's sample size and the *F*_ST_ values reported, with smaller studies reporting slightly higher *F*_ST_ values (*r*^2^ = 0.0049, *p*-value < 0.001, electronic supplementary material, figure S3). This should not significantly affect our analyses, however, given all of them are weighted by the average sample size of each study, giving less weight in the regression to smaller studies.

**Figure 2.**
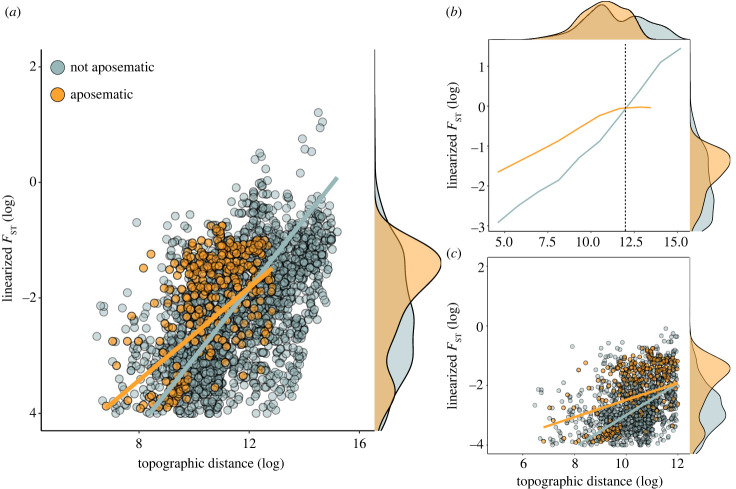
(*a*) Association between topographic distance and genetic differentiation (*F*_ST_) for aposematic (21 spp.) and non-aposematic lineages (43 spp.). The graph shows raw values for the microsatellite dataset (69% of data) and slope prediction from the full model presented in figure 3*a*. Each point represents a pair of populations. (*b*) Smooth functions predicted from GAMM analysis for the microsatellite data. For aposematic lineages the linear relationship is lost after a log distance value of 12 (162 km). (*c*) Similar to graph in (*a*) but using only the dataset up to a log distance of 12; model predictions correspond to GLMM reported in figure 3*b*. Plots along the right axes of all three graphs (and along the top border of graph *b*) represent density distributions.

**Figure 3.**
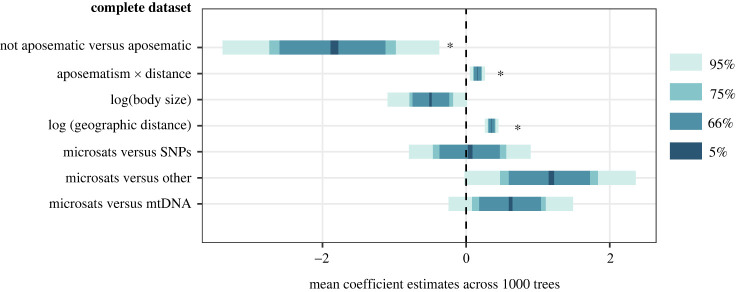
Graphic summary of GLMMs results using the complete anuran dataset. Confidence intervals of the estimate (effect) were calculated from the posterior probability distribution for the mean coefficient estimates across 1000 phylogenetic hypotheses. 95% intervals that do not overlap with zero are highlighted with an asterisk. Negative estimates are associated with a decrease in genetic divergence. Numerical results presented as electronic supplementary material, table S1 and the reduced dataset presented in electronic supplementary material, figure S5 and table S2.

Topographic distance strongly predicted the level of genetic differentiation between populations, as expected (figure 2*a*, electronic supplementary material, table S1). We found a significant interaction between aposematism and topographic distance. At shorter distances, species that were classified as aposematic presented significantly higher levels of genetic differentiation (higher *F*_ST_ values) relative to non-aposematic species, after considering the effect of distance and other variables (figure 2*a*). However, *F*_ST_ values for aposematic and non-aposematic species converged at longer distances. Body size had only a marginal effect on genetic differentiation and there were no significant differences across genetic markers (electronic supplementary material, table S1). The GAMM analysis showed similar results, but showed a saturation point for the aposematic dataset at around 162 km (12 log distance, figure 2*b*). This could be due to low sampling at high distances for that subset of species. Alternatively, it is possible that at such high distances gene flow between populations is effectively zero, so genetic differentiation stops increasing. When using a reduced dataset that included only the linear association between *F*_ST_ and distance for all species (distance values below 12, 60% of data), we found qualitatively identical results as in the full model (figure 3 and electronic supplementary material, figure S5 and table S2).

**Figure 4.**
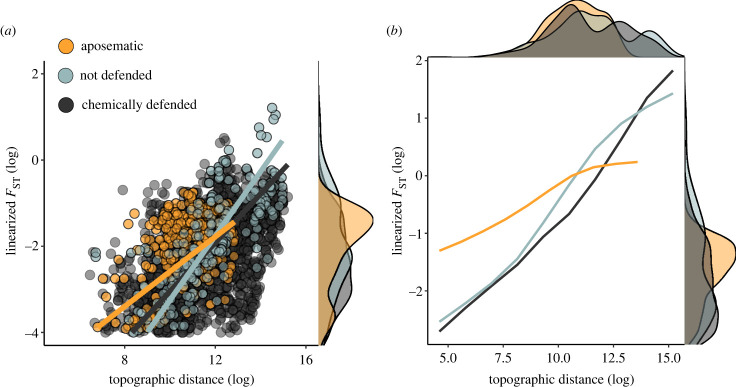
Association between distance and genetic differentiation using microsatellite data and three categories of anti-predator strategy: aposematic species, chemically defended species (but not conspicuous) and non-chemically defended species. (*a*) Raw data and estimates from GLMM presented in electronic supplementary material, table S3. (*b*) Predictions from GAMM. Plots on the right axes of each graph represent density distributions.

When anti-predator strategies were recategorized into aposematic, chemically defended and non-defended species, we found similar patterns as those reported above. Aposematic species had higher levels of genetic differentiation compared with non-defended species and marginally higher levels than chemically defended species (electronic supplementary material, table S3). Levels between non-defended species and only chemically defended species were similar (figure 4). Latitude had no significant effect on genetic differentiation in any of the models that included latitude as a predictor (electronic supplementary material, table S5, electronic supplementary material, figure S7). When only the subset of tropical species was used there was still a significant effect of the interaction between aposematism and distance, with stronger differences at shorter distances, but the independent effect of aposematism was lost (electronic supplementary material, table S5).

## 4. Discussion

Using a meta-analytical approach, we tested whether species that exhibit warning signals as anti-predator defence (aposematism) accumulated higher levels of genetic differentiation compared with species that are not aposematic, after considering the effects of distance and other variables. Our results show that populations separated by larger distances had higher levels of genetic differentiation and that, given the same topographic distance, populations of aposematic species are more likely to accrue higher levels of genetic differentiation. The effect of distance on genetic differentiation (i.e. the slope of the relationship between topographic distance and genetic differentiation), however, is weaker for aposematic species, and the potential effect of an aposematic strategy—that is, the difference between aposematic versus non-aposematic species—tends to be stronger at shorter topographic distances. Taken together, our results suggest that warning signals might be associated with reduced gene flow between populations, at least when distances between populations are small. This provides a mechanism that could potentially explain the high speciation rates previously detected in aposematic lineages [9,10] and verified in our own dataset (electronic supplementary material, figure S4).

Different studies have found a link between the use of warning signals as an anti-predator strategy and high speciation rates or species richness [8–10]. Anti-predator defences are posited to provide an escape from the evolutionary pressures of predation and result in increased ecological and evolutionary success [1,7,51,52]. The micro-evolutionary mechanisms underlying the observed link between the anti-predator defence and speciation, however, are far from clear. High speciation rates could result from various micro-evolutionary processes [53,54]. For example, speciation rates could increase due to ecological divergence, which could occur in sympatry [55], or colonization of novel environments could increase opportunities for speciation [56–58]. One of the most common demographic controls of high speciation rates, however, is geographical isolation [53], and there are several examples of lineages where restricted dispersal is linked to decreased gene flow and higher speciation rates [12,14,59]. Our results show that at a small scale (i.e. short distances), aposematic lineages present lower levels of gene flow between populations, and support the idea that high speciation rates could be linked to restricted dispersal between populations of aposematic species.

Higher levels of genetic differentiation between populations of aposematic species could directly result from the frequency-dependent nature of aposematism. The fitness of aposematic prey increases with density [18,19], meaning that colonization of novel environments (or any area with low population density) could be less likely in aposematic lineages. Field studies have also shown that local colour phenotypes in aposematic species (familiar to predators) suffer lower predation rates compared with novel phenotypes [60–62]; although see [31,63,64]. Aposematism could restrict dispersal between populations of the same species that have diverged phenotypically. In fact, within the polytypic poison frog *O. pumilio*, Wang & Summers [65] showed that there was higher genetic structure between phenotypically dissimilar populations. Their results supported a model where phenotypic divergence between populations led to reduced gene flow through selection against immigrant phenotypes. Similarly, spot pattern in nudibranchs can predict genetic structure, with restricted gene flow between populations that look less similar [66]. We note, however, that some studies have found no evidence for an association between colour and genetic structure in Dendrobatid species (e.g. [67,68]).

One of the more consistent results across all subsets of analyses was the significant interaction between aposematism and topographic distance. Our analysis shows that the effect of topographic distance on genetic differentiation is weaker for aposematic species. This pattern could be expected in systems where other factors contribute significantly to genetic differentiation besides distance (i.e. a weaker signal of isolation by distance (IBD)). It is possible that aposematism is an important mechanism restricting gene flow but only if populations are relatively close. For populations that are farther apart, other larger scale processes, such as isolation by distance, could better explain genetic differentiation levels—leading to smaller differences between aposematic and non-aposematic lineages when populations are separated by longer distances. Most frog species have relatively limited dispersal abilities—one review found an average maximum dispersal distance of about 4.5 km across 19 species [69]—so genetic connectivity over much longer distances could reflect gene flow over multiple generations and potentially through intermediate populations. This could effectively disrupt any effect that aposematic coloration could have on genetic differentiation. It is also possible that our analysis lacked statistical power to detect differences in genetic differentiation when populations were separated by large distances. In our dataset, we found that maximum distances between populations of aposematic species were lower than those between non-aposematic species. Aposematic populations were always separated by less than 850 km, while 11% of the populations of non-aposematic species were separated by larger distances. Lower sampling at larger distances could explain the differences in slope we detected. Moreover, if we assume that sampling of populations in both categories was random, then the sampling difference in distances could also reflect smaller ranges in aposematic species. It would be interesting to test whether there are differences in the evolution of range sizes between aposematic species and species that do not employ this anti-predator strategy.

We found no differences between chemically defended and non-defended lineages, even though chemically defended lineages were previously found to exhibit higher speciation rates in general [9]. There was also a tendency for chemically defended species to have lower genetic differentiation than aposematic species (electronic supplementary material, table S3), suggesting that toxicity on its own is unlikely to lead to higher divergence, and it is the combination of toxicity and colour (aposematism) that is linked to higher differentiation (figure 4). Another potential scenario that could explain low levels of gene flow between aposematic populations (and selection against immigrants) is that aposematic lineages could be more likely to achieve other prezygotic reproductive barriers due to local assortative mating. Sexual selection based on colour and assortative mating have been reported in poison frogs [70–74], and this could also be a mechanism that restricts gene flow between populations. Colour in Dendrobatidae has been proposed to be a ‘magic trait’ linked to speciation via both natural and sexual selection [61,70,75]. In fact, previous work found that aposematic lineages experienced higher speciation rates compared with non-aposematic lineages within the family Dendrobatidae [10], suggesting that it is aposematism, and not other traits shared across the family, driving increases in diversification rates. The low levels of gene flow between populations of aposematic lineages that we detected could thus be a product of not only predator selection against migration but also assortative mating within populations and sexual selection acting against novel phenotypes.

Another consideration is that polymorphic species have been suggested to have higher speciation rates [76,77] and might also be more likely to have genetically structured populations [31,67]. Although polymorphism specifically refers to variation within populations, variation in colour is a widespread phenomenon in frogs, and some of the best examples are aposematic frog species. In our dataset, there were several species known to exhibition variation in aposematic signals across populations (e.g. *Oophaga pumilio*, *Adelphobates galactonotus*, *Atelopus varius*, *Oophaga sylvatica*). Nevertheless, we do not think our results were driven by variable aposematic species with high genetic differentiation, because results were qualitatively identical even if we removed these species from the dataset (electronic supplementary material, table S4). Our dataset is not extensive enough, though, to test whether variable aposematic species (*n* = 8) tend to have higher population structure than monomorphic species, but this idea could be tested in future studies. Furthermore, it is also difficult to accurately classify species as polymorphic or polytypic. For instance, Klonoski *et al.* [78] suggest that *Mantella aurantiaca* and *M. crocea* could be considered either two separate species or two morphs of the same species. Something similar occurs with *O. lehmanni* and *O. histrionica*, which are known to hybridize in the field but maintain species status [71,79]. We also note that there could be other factors contributing to higher genetic divergence in aposematic species that are not related to the anti-predator strategy of aposematism. For example, many of the aposematic species we have in our dataset live in mountain chains, such as the Andes in South America or the Great Dividing Range in Australia. Distribution in mountains could potentially contribute to higher divergence, even though the distance measures we employed do consider topography. In the future, a larger sampling of aposematic species would allow us to separate the effects of these two factors.

Our results also support the general notion that speciation is more likely when there is geographical isolation and restricted gene flow between populations [53]. A positive link between genetic differentiation and speciation rates has been shown in lineages such as birds and fish [13,80]. However, despite being extensively predicted by theory, there is no evidence of such link in orchids, sea snakes or reptiles in general [81–84], suggesting that in some lineages, other processes independent of genetic differentiation might promote or limit reproductive isolation. To our knowledge, no studies have explicitly tested for a link between genetic differentiation and speciation rates in amphibians, but our results offer indirect evidence. Similar to birds [13], genetic differentiation among anuran populations could be tied to the processes that underlie macroevolutionary patterns of diversity in this clade. Additionally, although aposematic species were more often distributed in tropical latitudes, we did not find any evidence that latitude was linked to genetic differentiation, or that tropical species had higher genetic divergence.

To conclude, we uncovered evidence that aposematism may be linked to reduced dispersal and higher genetic differentiation between populations of frogs based on a meta-analysis of 64 species. This link could potentially be a mechanism contributing to the high speciation rates previously reported in aposematic lineages. Contrary to the notion that aposematism could facilitate colonization of new environments, our results suggest that this frequency-dependent strategy could restrict movement of individuals in populations separated by short distances, and increase the likelihood of divergence. Future studies could tease apart the ecological processes behind restricted gene flow in these species and compare, for example, whether cryptic morphs of species considered to be aposematic are able to disperse more effectively, colonize new territories more readily, and reproduce as migrants more frequently compared with aposematic morphs.

## Data Availability

The datasets and code used during the current study are available from the Dryad Digital Repository: https://doi.org/10.5061/dryad.fxpnvx0zk [85]. Extended methods, tables and figures are provided in the electronic supplementary material [86].
